# Quantification of dendritic and axonal growth after injury to the auditory system of the adult cricket *Gryllus bimaculatus*

**DOI:** 10.3389/fphys.2012.00367

**Published:** 2013-08-23

**Authors:** Alexandra Pfister, Amy Johnson, Olaf Ellers, Hadley W. Horch

**Affiliations:** ^1^Department of Invertebrate Zoology, American Museum of Natural HistoryNew York, NY, USA; ^2^Department of Biology, Bowdoin CollegeBrunswick, ME, USA

**Keywords:** sexual dimorphism, anatomical plasticity, midline guidance

## Abstract

Dendrite and axon growth and branching during development are regulated by a complex set of intracellular and external signals. However, the cues that maintain or influence adult neuronal morphology are less well understood. Injury and deafferentation tend to have negative effects on adult nervous systems. An interesting example of injury-induced compensatory growth is seen in the cricket, *Gryllus bimaculatus*. After unilateral loss of an ear in the adult cricket, auditory neurons within the central nervous system (CNS) sprout to compensate for the injury. Specifically, after being deafferented, ascending neurons (AN-1 and AN-2) send dendrites across the midline of the prothoracic ganglion where they receive input from auditory afferents that project through the contralateral auditory nerve (N5). Deafferentation also triggers contralateral N5 axonal growth. In this study, we quantified AN dendritic and N5 axonal growth at 30 h, as well as at 3, 5, 7, 14, and 20 days after deafferentation in adult crickets. Significant differences in the rates of dendritic growth between males and females were noted. In females, dendritic growth rates were non-linear; a rapid burst of dendritic extension in the first few days was followed by a plateau reached at 3 days after deafferentation. In males, however, dendritic growth rates were linear, with dendrites growing steadily over time and reaching lengths, on average, twice as long as in females. On the other hand, rates of N5 axonal growth showed no significant sexual dimorphism and were linear. Within each animal, the growth rates of dendrites and axons were not correlated, indicating that independent factors likely influence dendritic and axonal growth in response to injury in this system. Our findings provide a basis for future study of the cellular features that allow differing dendrite and axon growth patterns as well as sexually dimorphic dendritic growth in response to deafferentation.

## Introduction

Anatomical plasticity in the adult central nervous system (CNS) is presumed to be important for successful recovery from serious neuronal injuries. However, only a handful of examples of such plasticity in the CNS in either adult vertebrates or invertebrates have been described, mainly in a few different species or in particular injury scenarios (Murphey et al., 1975; Bulloch and Ridgway, 1989; Büschges et al., 1992; Wolf and Büschges, 1997; Krüger et al., 2011b; Tavosanis, 2012). One of the more striking, positive, compensatory responses to injury has been described in the auditory systems of several species of field crickets (Hoy et al., 1985; Schildberger et al., 1986; Brodfuehrer and Hoy, 1988). Crickets have evolved a mechanism to compensate for injury to the auditory system, a sensory system crucial to cricket survival and reproduction, and because this compensatory response is unusual for any species in the animal kingdom, it is worthy of in-depth study.

The compensatory response in the cricket is complex. It involves both sprouting of deafferented dendrites and of intact, contralateral axons as a direct and indirect result of deafferentation, respectively, as well as novel synapse formation and functional recovery. Unlike the cricket, many denervation experiments in embryonic and developing animals typically result in post-synaptic neuronal decline and death (Parks, 1979; Trune, 1982a,b; Nordeen et al., 1983; Born and Rubel, 1985) while denervation in mature animals minimally affects post-synaptic neurons (Born and Rubel, 1985; Hashisaki and Rubel, 1989; Moore, 1990). The general conclusions derived from these studies are that pre-synaptic input is necessary for growth and stabilization of their post-synaptic partners during development, but once neuronal systems mature in the adult, many classes of neurons reach a stable state and do not need active input to maintain their structure (Tavosanis, 2012).

Denervation-induced withdrawal and death does not occur in all systems, and intriguing exceptions have been identified in both vertebrates and invertebrates. For example, deprivation of sensory input in adult rodents, via whisker removal or forepaw denervation, leads to indirect denervation of cortical neurons and has been shown to induce dendritic reorganization (Hickmott and Steen, 2005; Tailby et al., 2005). However, additional studies have failed to detect large-scale reorganization of cortical dendrites after visual deprivation (Hofer et al., 2008) or even after direct retinal damage (Keck et al., 2008) in the adult. Physical injury to neurons via axotomy can lead to varying amounts of dendritic and axonal growth as well as shifts in dendritic arbor organization, as is seen in mouse superior cervical ganglion cells (Yawo, 1987), lamprey central neurons (Hall and Cohen, 1983), feline neck motor neuron (Rose et al., 2001), cricket terminal ganglion interneurons (Chiba et al., 1988; Chiba and Murphey, 1991), and in the cricket and locust auditory systems (Pallas and Hoy, 1986; Lakes and Kalmring, 1991; Krüger et al., 2011b). Deafferentation-induced elevator motor neuron sprouting in the metathoracic ganglion of locusts helps these animals recover proper wing patterns after unilateral tegula removal (Büschges et al., 1992; Wolf and Büschges, 1997). Within the cricket, deafferentation-induced compensatory dendritic responses have been demonstrated in both the terminal ganglion and the prothoracic ganglion (Murphey et al., 1975; Murphey and Levine, 1980; Hoy et al., 1985; Pallas and Hoy, 1986; Schildberger et al., 1986; Brodfuehrer and Hoy, 1988; Schmitz, 1989; Kanou et al., 2004; Horch et al., 2011; Krüger et al., 2011a,b). The deafferentation-induced compensation in the terminal ganglion consists mainly of changes in the strength of existing synapses (Murphey and Levine, 1980), though some dendritic sprouting has been noted (Murphey et al., 1975). The deafferentation-induced functional recovery in the auditory system of the cricket, on the other hand, is a result of extensive post-synaptic sprouting and reorganization as well as sprouting from intact, contralateral axons in both juveniles and adults (Schmitz, 1989; Horch et al., 2011). It is notable that functional recovery in the auditory system appears to require that axons and dendrites grow across the midline, a landmark that typically serves as an inhibitory boundary (Pallas and Hoy, 1986; Brodfuehrer and Hoy, 1988; Schmitz, 1989).

Adult crickets detect sound waves with tympanal membranes and associated auditory organs that are located on the tibial section of the cricket's forelegs. Auditory information is conveyed to the prothoracic ganglion in the CNS via nerve 5 (N5), and the auditory receptor axons end in a claw-shaped arbor within the medioventral association center (mVAC). Auditory interneurons located in the prothoracic ganglion are arranged in bilaterally symmetric pairs on either side of the midline and receive their primary auditory information from the ipsilateral N5. Individual, identified auditory interneurons respond specifically to either the calls of male conspecific crickets [Ascending Neuron-1 (AN-1) responds best to 5 kHz], or the ultrasound pulses of predatory bats [Ascending Neuron-2 (AN-2) responds best to 15 kHz and higher]. These responses are shaped by the inhibitory input received by the ANs that is thought to come from the paired omega neurons (Hardt and Watson, 1994). N5 tonotopy is arranged such that afferents carrying low frequency information to AN-1 are tightly clustered in the most anterior medial portion of N5 where they meet densely clustered AN-1 dendrites. N5 afferents carrying ultrasound information are somewhat more diffuse and primarily lie along the medial edge of the anterior portion of the N5. AN-2 dendrites in turn span the medial edge of N5. Low and ultrasound frequency variscosities overlap in some areas of N5 (Wohlers and Huber, 1985; Imaizumi and Pollack, 2005). N5 carries additional axons into the prothoracic ganglion that originate in additional sensory organs. Many of these axons arborize more laterally, though a small group arborizes in the peripheral region of the mVAC (Nishino and Sakai, 1997; Nishino, 2000). Upon removal of one foreleg, which results in the removal of the auditory organ and the deafferentation of the auditory interneurons on that side, these auditory interneuron dendrites in both larvae and adults sprout across the midline, forming functional synapses with the contralateral auditory nerve (Hoy et al., 1985; Schildberger et al., 1986; Brodfuehrer and Hoy, 1988). The majority of crickets deafferented as adults regain responsiveness to auditory stimulation in the deafferented interneurons between 4 and 6 days after ear removal, and physiological recordings demonstrate a partial recovery of cell-type-specific auditory responses (Brodfuehrer and Hoy, 1988).

Few studies have quantified anatomical characteristics of the compensatory growth after deafferentation. Previously, our group quantified bilateral adult auditory anatomical changes induced by chronic deafferentation throughout larval development (Horch et al., 2011). However, the quantification of the anatomical changes induced by deafferentation in the adult has been explored in only a few studies (Brodfuehrer and Hoy, 1988; Schmitz, 1989). The Schmitz study (1989) describes deafferented AN-2 dendrites approaching and crossing the midline by about 7 days and reaching their maximum extent by 27 days. This study also demonstrates that the number of contralateral N5 axons projecting across the midline is significantly greater in deafferents than in controls but that the distance the N5 axons extend over the midline is not significantly different between controls and deafferented animals. These results identify general growth trends of AN-2 and N5 after deafferentation in females (Schmitz, 1989), but fine time-scale measurements were not made and male data were not collected. Thus, it is currently unclear if male and female growth responses differ. Furthermore, the precise timeline of the growth responses in three dimensions has never been examined.

In the present study we focused on quantifying three-dimensional changes in N5 axons and in the dendrites of ANs over time. Modern neuronal tracers and three-dimensional reconstructions of confocal images were used to trace and measure AN dendrites and N5 axons. Control *Gryllus bimaculatus* N5 and AN morphology were compared to that seen after 30 h as well as after 3, 5, 7, 14, and 20 days of deafferentation. Growth patterns were statistically analyzed, and here we report the most detailed account thus far of deafferentation-induced compensatory growth over time. Most intriguingly, we demonstrate that female AN dendrites grow at significantly different rates than male dendrites and differently from axons in either males or females.

## Methods

### Subjects

*Gryllus bimaculatus* (originally supplied by Ron Hoy, Cornell University) were raised in a 12:12 light:dark cycle at 28°C in 40–60% humidity. They were fed cat chow and water *ad libitum*. For neuroanatomical studies both male and female adult crickets, no more than 1 week past adult molt, were used. After cooling, one prothoracic leg was removed proximal to the tympanal membrane. Right and left leg deafferents were prepared in roughly equal numbers, and since no differences were found, all left/right data were combined. Control crickets and those deafferented for 30 h, 3, 5, 7, 14, or 20 days were grouped by deafferentation length and housed under the same conditions as above.

### Backfilling cricket ascending neurons and contralateral nerve 5

Backfills were performed as described by Horch et al. (2011). Briefly, backfills were performed “*in situ*” in cooled, immobilized crickets. Neck connective axons and nerve 5 axons were separately submerged in Vaseline wells containing one of two different neuronal tracers. A small number of axons, including the AN axons, were filled with 4% unconjugated biocytin (Sigma-Aldrich, St. Louis, MO, USA) in 50 mM NaHCO_3_ (American Bioanalytical, Natick, MA, USA). N5 axons, those contralateral to the amputation in deafferented animals, were backfilled with 1 mg/μL biocytin conjugated to Alexa Fluor 594 (Invitrogen, Carlsbad CA, USA) in cricket saline (140 mM NaCl, 5 mM KCl, 7 mM CaCl2-2H2O, 1 mM MgCl2-6H2O, 5 mM TES, 4 mM NaHCO3, 5 mM trehalose, pH 7.3). Dye was allowed to be transported at 4°C for approximately 16–20 h, after which tissue was fixed and biocytin was visualized using 1:400 streptavidin conjugated to Alexa Fluro 488 (Invitrogen, Carlsbad, CA), as described by Horch et al. (2011). Tissue was rinsed, dehydrated through an ethanol series, and mounted, on a slide with coverslip spacers, ventral side up in methyl salicylate.

### Confocal microscopy

Backfilled ganglia were treated with streptavidin and imaged with a Zeiss LSM510 META laser scanning confocal microscope (Thornwood NY, USA). Three-dimensional images of ANs and N5 were taken with a Plan-NEOFLUAR 10X/0,30 and the Plan-NEOFLUAR 40X/1,3 objectives using the 488 nm and 543 nm lasers. For greater magnification, images were visualized under the 40× objective with a digital 2× zoom. To capture the location of the midline, the 633 nm laser was used to collect corresponding 40× images of the autofluorescent cells at the midline.

### AN and N5 anatomical quantification

AN and N5 growth across the midline were analyzed and quantified “blindly” in coded images using Volocity High Performance 3D Imaging Software (PerkinElmer, Waltham MA, USA). Autofluorescent glial cells collected with 633 nm laser were a reliable marker of the midline, which was represented by a single line running through the glial cells (shown in blue, Figures 2A,B). This line was then transferred, based on coordinate locations, to images of ANs and N5 (Figure 2C).

Using Volocity to scroll through sequential planes, axonal and dendritic branches were traced by hand in three dimensions. AN and N5 processes were quantified by the perpendicular extent of the point furthest from the midline (perpendicular extent), by the longest dendrite, and by skeletal length (the summed length of all the processes; Figure 2D). The volume of axons that project outside the typical medial ventral association center (mVAC) region was also measured. The upper region of the N5 “claw-shaped” arborization has a distinct posterior edge in control crickets that was approximated by a straight line for analysis. The volume of axons that projected across this line or across the mid-line was measured using Volocity (Figure 5).

### Data analysis

The tracing measurements and volumetric data sets were analyzed using the non-parametric Spearman rank correlation to determine whether there were significant trends in the data. When there were significant trends in the data, data were fit with linear and non-linear fitting techniques. Standard linear regression techniques were used for linear fits:

(1) y=mx+b

where *y* is the variable of interest, *x* is time in days, *m* is the slope of the line and *b* is the intercept; *m* and *b* are fitted terms. Non-linear fits were done by using a standard von Bertalanffy growth function, an equation commonly used to describe growth rates over time:

(2) $$y\text{= }l_{\infty }\text{−}\left(l_{\infty }\text{− }l_{0}\right)\;e^{\text{−}k\;x}$$

where *y* is the variable of interest, *x* is time, *l*_∞_ is the ultimate value of *y, l*_0_ is the value of *y* at time = 0 and *k* is a rate of change; *l*_∞_, *l*_0_, and *k* are fitted terms.

The Akaike information criterion corrected model (AICc) was used to compare whether to select the non-linear or linear fit to the data. If the probability given by AICc that the linear model was correct was greater than 50% then the linear model was selected; if the probability that the linear model was correct was less than 40% then the non-linear model was selected; otherwise the preferred model was considered “undetermined.”

For all data the end-point of measured variables for males versus females was statistically compared; *t*-tests were used when the data met the assumptions (normality and equality of variances) of the parametric *t*-test, otherwise a non-parametric Kruskal–Wallis test was used for the end-point comparisons. In addition, an ANCOVA was used to compare genders if neither of the genders were better fit by the non-linear fit and if at least one of the genders had a significantly non-zero slope when fit with linear regression analysis. A non-parametric Kruskal–Wallis test on all the data was used to compare genders when there were no linear trends in the data. Within-animal AN skeletal length was compared with N5 skeletal length, N5 extraneuropillar growth, N5 extraneuropillar growth at 5 and 7 days, and Male N5 extraneuropillar growth using Spearman rank correlation to determine whether there were significant correlations in the growth of AN and N5.

## Results

**Figure 1:**
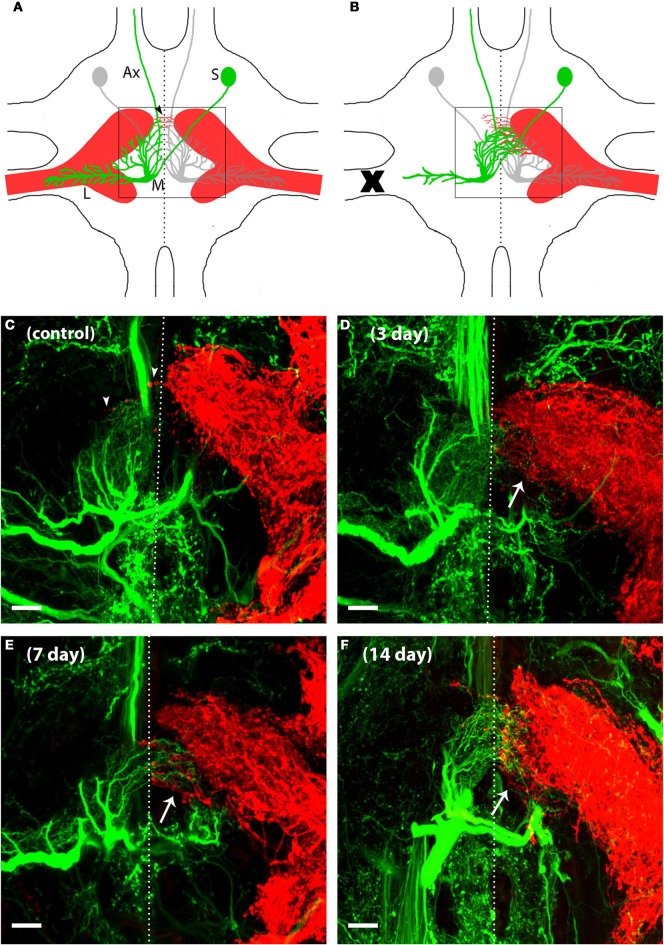
**AN dendrites and N5 axons reorganized their neuronal structure to grow toward one another after deafferentation in adult crickets.** N5 (red) is composed in part of axons that carry auditory information from the tympanal organ on the cricket's forelegs to the prothoracic ganglion where a variety of neurons, including AN-2 (green), receive the auditory information and send this information to the brain. **(A)** The schematic of a prothoracic ganglion in a control animal shows that AN-2 as well as N5 are bilaterally symmetric. The N5 axonal terminal structure resembles a “claw” shape. AN-2 dendrites are arranged in an L shape, with medial dendrites (M) synapsing with the upper portion of the ipsilateral N5 claw and lateral dendrites (L) extending into the N5 axonal track. Postsynaptic information is relayed to the brain via the AN-2 axon (Ax) that extends through the anterior end of the prothoracic ganglion. The AN-2 soma (S) is located on the contralateral side of the midline from its dendrites. In control animals a few N5 axons may extend across the midline (arrowheads) while AN-2 dendrites tend to respect the midline. **(B)** After one foreleg is removed (X) in an adult cricket, deafferented AN medial dendrites grow across the midline toward the contralateral N5. In addition, N5 axons on the intact side extend toward the deafferented AN dendrites. Only a small portion of total post-deafferentation N5 growth crosses the midline (arrows indicate the area where the majority of N5 compensatory growth emerges). 40× z-stack confocal images of **(C)** control, **(D)** 3 days, **(E)** 7 days, and **(F)** 14 days deafferents display general AN and N5 anatomy and increasing amounts of growth following deafferentation. In order to see clearly the dendrites at the midline, a subset of this cell's optical sections were projected. The lateral dendrites of this cell were in optical sections that were not included in this projection. The dotted lines represent approximated midlines. Scale bars = 20 μm.

Backfills using neuronal tracers allowed us to visualize AN dendrites (for simplicity, only AN-2 is shown in green) and N5 axons (red) in the same prothoracic ganglia in control adult crickets (Figure 1). Our backfills typically result in robust fills of AN-2, but sometimes include weak or incomplete backfills of AN-1 as well. Given the close proximity and overlap of AN-1 and AN-2 dendrites, we will refer to these dendrites more generally as “AN” dendrites throughout. As has been established for this system (Hoy et al., 1985; Schmitz, 1989; Horch et al., 2011), the AN dendrites from a single cell remain largely on one side of the ganglion and do not cross the mid-line in significant numbers. The majority of N5 axons also roughly respect the midline, though as has been previously noted (Schmitz, 1989), several axons extend across the midline in many normal control animals (Figures 1A,C, arrowheads). Our double backfills confirm this anatomical arrangement in the prothoracic ganglia of adult control crickets (Figure 1C).

To understand how deafferentation in adulthood altered dendritic and axonal growth over time, we deafferented adult animals and backfilled both N5 and the ANs after 30 h, 3, 5, 7, 14, and 20 days. Representative backfills of a subset of these time points are shown in Figures 1C–F. Qualitatively, AN growth increased fairly rapidly over the midline after deafferentation, and generally appeared to reach its maximum extent within a week. The amount of N5 axonal growth beyond the midline increased somewhat after deafferentation, with a few additional axons crossing the midline. More dramatic, however, was the substantial sprouting of N5 axons from the posterior edge of the anterior portion of the N5 “claw” (see arrows in Figures 1B–E, and Horch et al., 2011).

### Quantification of an dendritic growth after deafferentation

**Figure 2:**
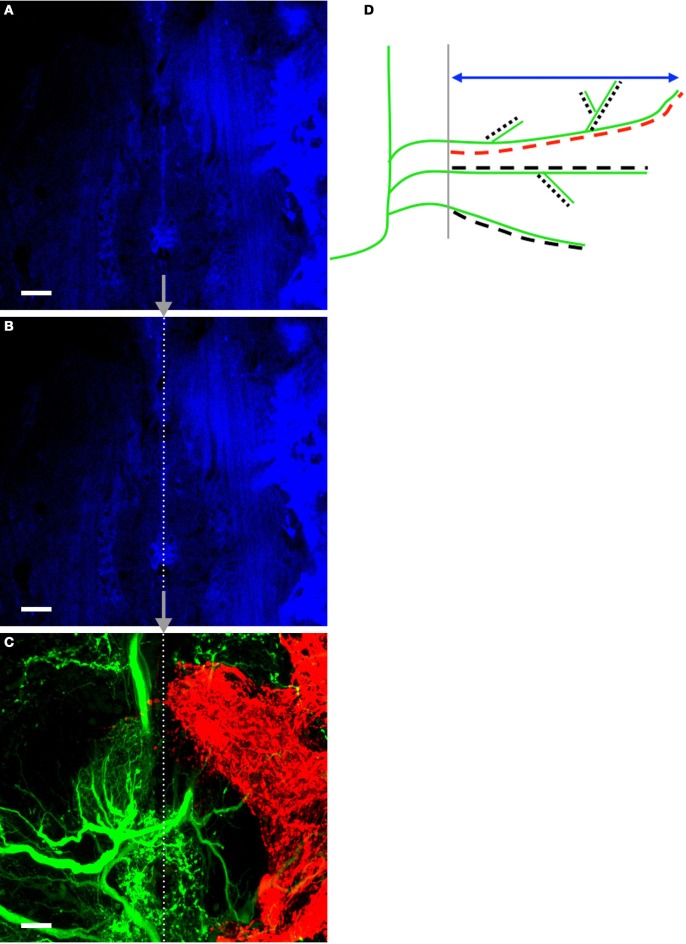
**Method for finding and placing the midline on tracing images and measurement schematic. (A)** Autofluorescent cells at the midline of the prothoracic ganglia were captured in a confocal image. **(B)** Prior to hand-tracing AN and N5 processes, the midline was approximated by tracing a straight line over the autofluorescent cells. **(C)** A corresponding image of the ANs and N5 was subsequently captured. The midline was traced onto the AN/N5 image by placing the end-points of the line at the exact coordinates as they appeared on the midline image. The images shown belong to a control cricket. The midline is represented by the dotted line. Scale bars = 20 μm. A schematic highlighting the measurements made in this study is shown in **(D)**. AN dendrites crossing the midline (gray) are represented in the schematic in green. The double-headed arrow (blue) indicates the perpendicular extent measurement. The longest dendrite length was recorded for each animal and is highlighted here in red. All dendrites and their branches were hand-traced (represented by the dashed lines), and the skeletal length measurement is the sum of the lengths of the dashed lines. For simplicity AN dendrites only are represented here but the same methods were used to measure N5 axonal growth across the midline.

To better understand the nature of this growth over time, we quantified a variety of axonal and dendritic characteristics after deafferentation. Accurately quantifying aspects of those axons and dendrites crossing the midline required that we first establish a consistent, unbiased method of identifying the midline. As noted in the methods, we developed a protocol in which we used the autofluorescence of the midline region in coded images (Figure 2A) to determine the midline location, which was then approximated as a straight line (Figure 2B). The extent to which axons and dendrites had crossed this midline was determined first by hand-tracing individual processes that had been backfilled with dye (Figure 2C) in control ganglia and in ganglia from each deafferentation period and then by taking a variety of measurements from these traces. The schematic in Figure 2D illustrates the measurements collected.

**Figure 3:**
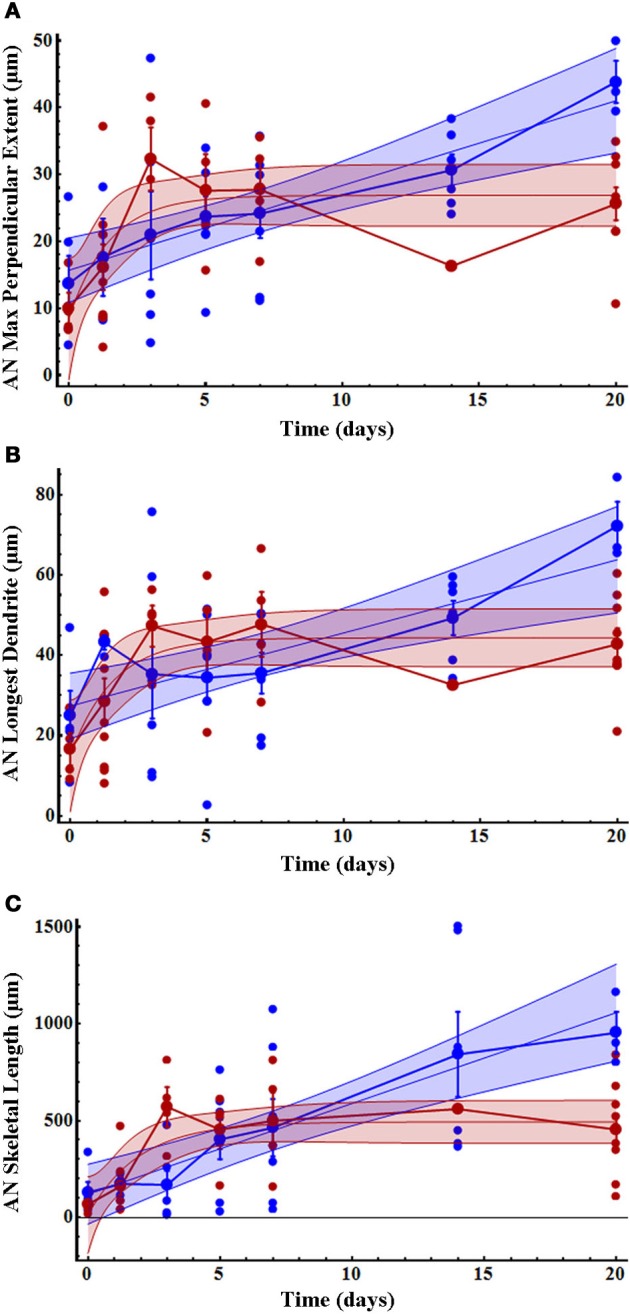
**Initial AN growth across the midline increased more rapidly in females than males over time after deafferentation. (A)** AN dendritic maximum perpendicular extent, **(B)** longest dendrite, and **(C)** skeletal length increased non-linearly in females (red) and linearly in males (blue). Dendritic length in females reached a plateau by 3 days after deafferentation, while male dendritic length increased more slowly and did not plateau within the measured time period. The mean for each time point is shown as a slightly larger data point, and error bars represent standard error of the mean. The blue and red shaded areas represent 95% confidence intervals. Female AN control (*n* = 4), 30 h (*n* = 9), 3 days (*n* = 4), 5 days (*n* = 4), 7 days (*n* = 7), 14 days (*n* = 1), and 20 days (*n* = 9). Male AN control (*n* = 5), 30 h (*n* = 4), 3 days (*n* = 6), 5 days (*n* = 8), 7 days (*n* = 7), 14 days (*n* = 6), and 20 days (*n* = 3). See Table 1 for regression equations and statistics.

A small number of AN dendrites normally extended a short distance across the midline in control crickets (*T* = 0, Figure 3A); however, dendrites in deafferented crickets rapidly extended further across the midline. AN growth patterns were significantly and strikingly different in male and female crickets. In males, the maximum perpendicular extent, the longest dendrite, and the skeletal length each increased linearly following deafferentation. In females, however, growth of each of these variables increased non-linearly and were characterized by a rapid growth phase in the first 3 days followed by a plateau in length (Figures 3A–C; Table 1). In general, female data were less variable than male data (Table 1). While at day 0 there were no significant differences in dendritic characteristics (*t*-tests, each df = 10, each *p* > 0.3; see also 95% confidence intervals of linear and non-linear fits), by day 20, all dendritic characteristics were 45–110% greater in males than in females (Table 1).

**Table 1:** **Summary of variables and statistics for AN growth analysis**.

**Variable**	**Gender**	**Spearman rank**			**Compare linear to non-linear**			***r*^2^ and fitted parameters *in* for preferred model**							***t*-test comparing end point**
		**Sample size**	***r***	**1-tailed *P* (df)**	**ΔAICc**	**Probability linear model is correct**	**Preferred model**	***r*^**2**^**	***k* (per day)**	***m* (μm per day)**	***l*_o_(μm)**	***b*(μm)**	***l*_∞_(μm)**	***p* slope = 0 (df)**	***p(df)*M?F**
AN maximum perpendicular extent	Male	37	0.61	<0.0001 (35)	2.4	0.77	Linear	0.39	na	1.3	na	15.7	na	<0.0001 (1,35)	0.003 (10) M > F
	Female	35	0.48	0.002 (33)	8.3	0.02	Non-linear	0.88	0.68	na	8.4	na	29.2	na	
AN longest dendritic extent	Male	37	0.47	0.002 (35)	2.4	0.77	Linear	0.32	na	1.8	na	27.3	na	0.0003 (1,35)	0.003 (10) M > F
	Female	35	0.48	0.002 (33)	8.2	0.02	Non-linear	0.89	0.64	na	14.9	na	44.3	na	
AN skeletal length	Male	37	0.65	<0.0001 (35)	2.0	0.73	Linear	0.46	na	46.9	na	119.4	na	<0.0001 (1,35)	0.007 (10) M > F
	Female	35	0.58	<0.0001 (33)	9.9	0.01	Non-linear	0.80	0.52	na	12.0	na	492.8	na	

Data were first analyzed using the non-parametric Spearman rank correlation to determine whether there were significant trends in each variable over time. When the Spearman rank indicated a significant correlation, the data were fit with a linear and non-linear model (Equations 1 and 2) and the Akaike Information Criterion corrected model (AICc) was used to determine the preferred model. The parameters for the preferred model are given, with units indicated in parentheses. In addition, when the linear model was preferred the probability that the best fitting line has a zero slope and the degrees of freedom of that test are given. Finally, end-points were compared via a t-test after testing assumptions of normality and equal variance.

### Quantification of N5 axonal growth after deafferentation

**Figure 4:**
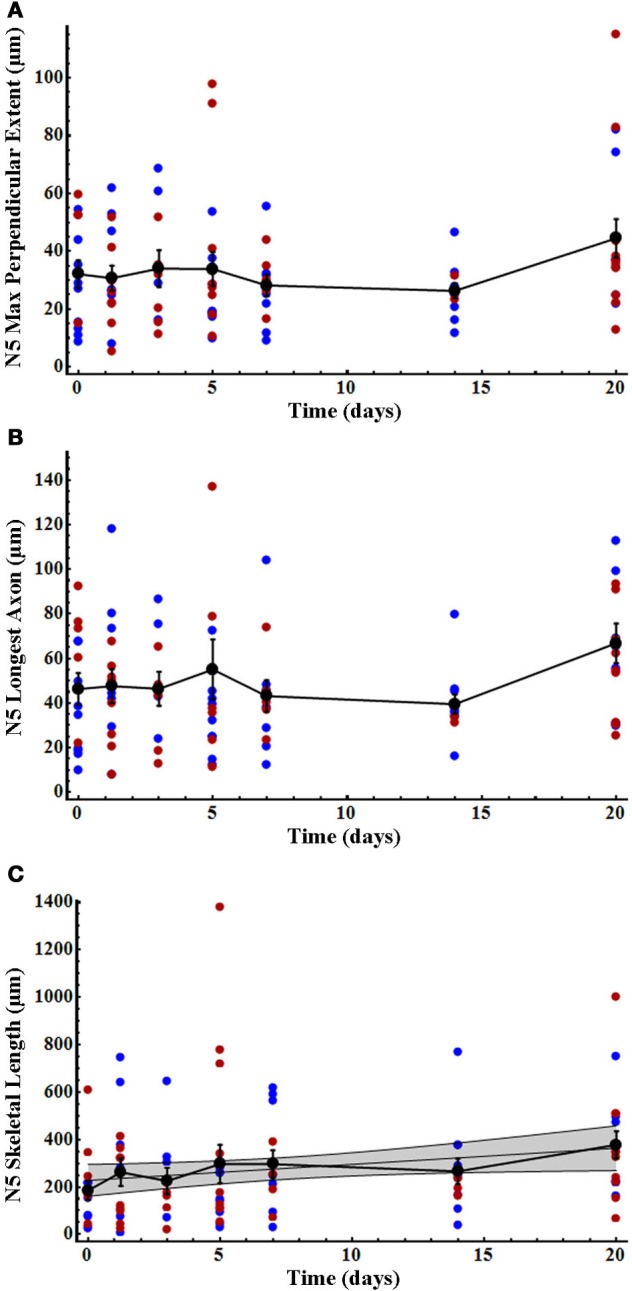
**N5 axonal skeletal length increased significantly over time after deafferentation, while the longest axon and perpendicular extent of N5 axons did not. (A)** N5 axonal maximum perpendicular extent and **(B)** longest axon showed no significant change over the deafferentation time period while **(C)** skeletal length increased significantly in a linear fashion. N5 growth patterns did not differ between males (blue) and females (red). Black data points represent means, and error bars represent standard error of the mean. The shaded gray area indicates the 95% confidence interval. Female N5 control (*n* = 6), 30 h (*n* = 9), 3 days (*n* = 6), 5 days (*n* = 9), 7 days (*n* = 5), 14 days (*n* = 3), and 20 days (*n* = 10). Male N5 control (*n* = 9), 30 h (*n* = 7), 3 days (*n* = 4), 5 days (*n* = 9), 7 days (*n* = 7), 14 days (*n* = 9), and 20 days (*n* = 7). See Table 2 for regression equations and statistics.

As previously noted, and as seen in the results of our present study, N5 axonal processes cross the midline in control animals far more frequently and more extensively than do AN-2 dendrites (Schmitz, 1989). However, N5 maximum perpendicular extent and longest axon did not change significantly after deafferentation over time (Figures 4A,B; Table 2), and there were no significant differences between males and females either specifically at the end-point of 20 days (Figures 4A,B; Table 2) or overall for data pooled over all time periods (Kruskal–Wallis tests, each df = 96, each *p* > 0.26). In contrast, N5 skeletal length increased linearly until day 20 for gender-pooled data (although not for male and female data analyzed separately: Figure 4C, Table 2) with no significant difference between skeletal length in males and females either at the end-point of 20 days (Table 2) or overall for data pooled over all time periods (Kruskal–Wallis test, df = 96, *p* = 0.93).

**Table 2:** **Summary of variables and statistics for N5 growth analysis**.

**Variable**	**Gender**	**Spearman rank**			**Compare linear to non-linear**			**r^2^ and fitted parameters *m*for preferred model**				***t*-test or Kruskal–Wallis comparing end point**
		**Sample size**	***r***	**1-tailed *P* (df)**	**ΔAICc**	**Probability linear model is correct**	**Preferred model**	***r*^**2**^**	***m* (μm per day)**	***b* (μm)**	***p* slope = 0 (df)**	***p* (df) M?F**
N5 max perpendicular extent	Male	52	0.101	0.24 (50)	na	na	na	na	na	na	na	0.93 (15) M = F (K–W)
	Female	46	0.094	0.27 (44)	na	na	na	na	na	na	na	
	Pooled	98	0.091	0.19 (96)	na	na	na	na	na	na	na	
N5 longest axon	Male	52	0.155	0.14 (50)	na	na	na	na	na	na	na	0.94 (15) M = F (*t*-test)
	Female	46	0.057	0.35 (44)	na	na	na	na	na	na	na	
	Pooled	98	0.102	0.16 (96)	na	na	na	na	na	na	na	
N5 skeletal length	Male	52	0.30	0.01 (50)	0.1	0.51	Linear	0.06	7.5	218.4	0.08 (1,50)	0.91 (15) M = F (*t*-test)
	Female	46	0.25	0.05 (44)	2.2	0.75	Linear	0.03	6.1	237.8	0.26 (1,44)	
	Pooled	98	0.27	0.003 (96)	2.1	0.74	Linear	0.04	6.8	227.7	0.04 (1,96)	
N5 extraneuropillar growth	Male	44	0.49	0.0003 (42)	2.3	0.76	Linear	0.24	710	6105	<0.001 (1,42)	0.52 (14) M = F (*t*-test)
	Female	46	0.36	0.008 (44)	0.13	0.52	Linear	0.11	461	9755	0.02 (1,44)	
	Pooled	90	0.42	<0.0001 (88)	1.6	0.69	Linear	0.17	579	7977	<0.0001 (1,88)	

For a description of the table columns, see the legend for Table 1 with the following addition: end-points were compared with a non-parametric Kruskal–Wallis test instead of a t-test when there were significant differences in variance around the male/female means; end-point data were always normally distributed.

**Figure 5:**
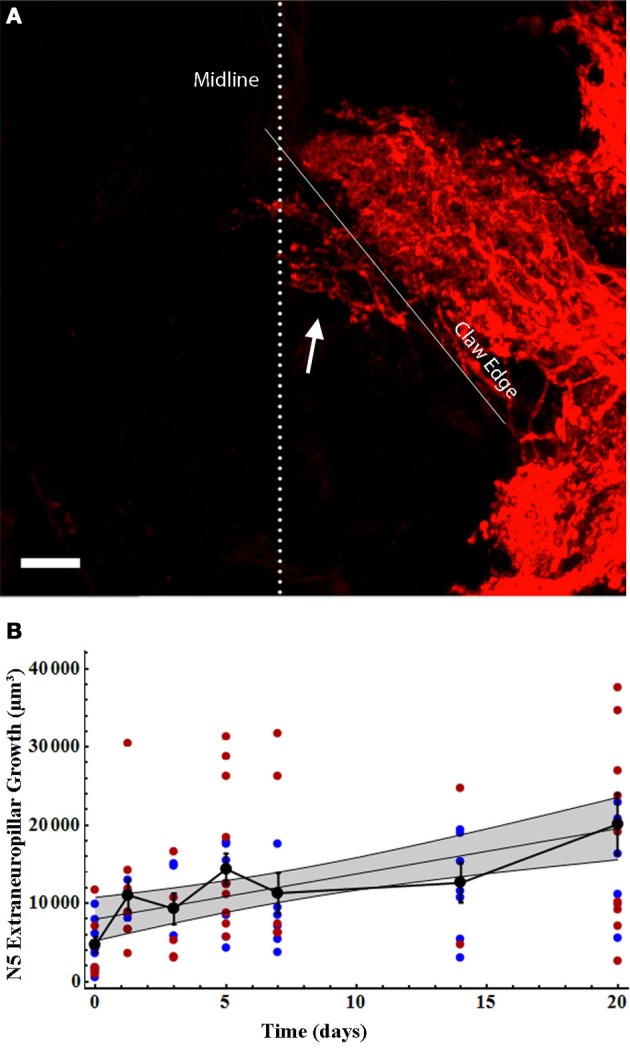
**N5 extraneuropillar growth increased in the same linear pattern for males and females following deafferentation. (A)** Total N5 growth consisted of the axons crossing the midline (dotted line) as well as the axons crossing the bottom edge of the anterior portion of the claw (solid white line) to extend outside of the neuropil (arrow). The volume of this extraneuropillar growth was measured. The N5 image shown belongs to a 7 days deafferent. Scale bar = 20 μm. **(B)** Male (blue) and female (red) N5 extraneuropillar growth data sets increased linearly and were not significantly different from one another. Black data points represent means, and error bars represent standard error of the mean. Female N5 extraneuropillar growth control (*n* = 7), 30 h (*n* = 6), 3 days (*n* = 5), 5 days (*n* = 10), 7 days (*n* = 6), 14 days (*n* = 2), and 20 days (*n* = 10). Male N5 extraneuropillar growth control (*n* = 9), 30 h (*n* = 6), 3 days (*n* = 3), 5 days (*n* = 7), 7 days (*n* = 6), 14 days (*n* = 7), and 20 days (*n* = 7). The shaded gray area represents the 95% confidence interval. See Table 2 for regression equations and statistics.

As we have previously described, many axons sprout from the N5 “claw” into the extra-neuropillar space, but do not cross the midline (Horch et al., 2011). As such, this axonal sprouting was not represented in our analysis above. This region of N5 growth, extending across the posterior edge of the anterior portion of the N5 claw, appears to be the location in which axons and contralateral ANs grow in close proximity and may be the locus for some of the functional recovery seen in deafferented AN neurons (Figures 1D–F, arrow, Horch et al., 2011). We quantified the volume of axon sprouting into this region (Figure 5A). Extra-neuropillar N5 sprouting increased linearly over time both in gender-pooled data and for male and female data analyzed separately (Figure 5B; Table 2), and there was not a significant difference between skeletal length in males and females overall for data pooled over all time periods (ANCOVA; *p*_equal slopes_: df = 86, *p* = 0.37; *p*_equal intercepts_: df = 87, *p* = 0.35).

### Axonal and dendritic growth were independent

**Figure 6:**
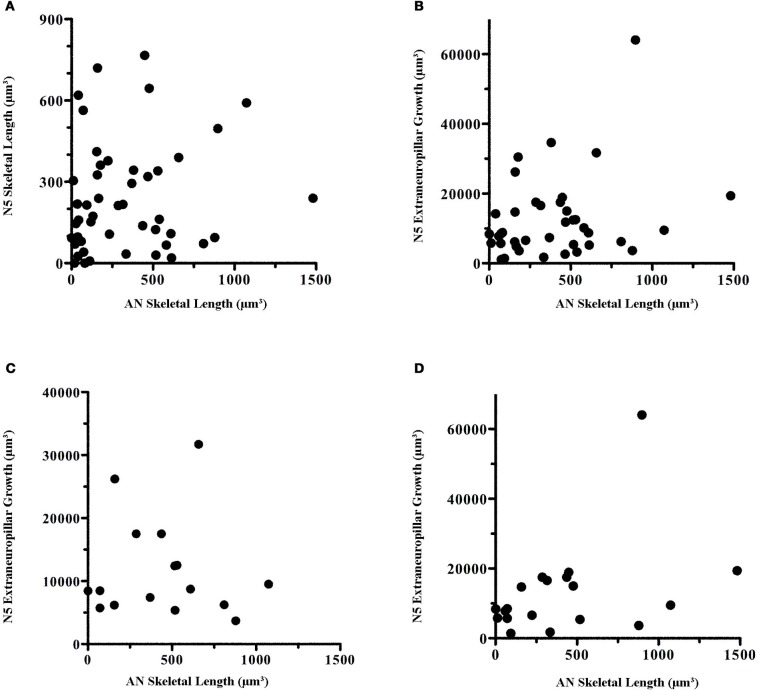
**There was no correlation in the amount of dendritic growth in comparison to axonal growth within individuals.** Within-animal AN skeletal length was compared with **(A)** N5 skeletal length, **(B)** N5 extraneuropillar growth, and **(C)** N5 extraneuropillar growth at 5 and 7 days. **(D)** Male N5 extraneuropillar growth at all time points also did not correlate with male AN skeletal length. These data were analyzed by Spearman rank correlation but there were no significant correlations among any of the comparisons (**A**: *p* = 0.16, *n* = 48; **B**: *p* = 0.21, *n* = 39; **C**: *p* = 0.88, *n* = 16; **D**: *p* = 0.10, *n* = 19).

Some of the variation in our data could have been due to variations among animals that possess inherent environments either more or less conducive to growth. If so, there should be a correlation between the extent of growth of axons and dendrites per animal, with some animals showing poor growth for both axons and dendrites and others exhibiting extensive growth for both axons and dendrites. There was, however, no correlation between axonal and dendritic growth within each animal (Figure 6). Even when comparing extraneuropillar axonal growth, arguably a more sensitive measurement of total axonal growth, with AN skeletal growth, no correlation was evident (Figure 6B).

Given that female AN growth rates were non-linear while axonal growth rates were linear, perhaps it is not surprising that there was no correlation between axonal and dendritic growth across the whole population over time. The early burst of growth at 3 days and the subsequent plateau for female dendrites would not correlate well with the steady, linear growth rates seen in axons. Thus, we examined the correlation between axonal and dendritic growth at 5 and 7 days. At these time points, male and female growth levels roughly overlapped (Figure 3). However, no correlation was evident between axonal extraneuropillar growth and AN skeletal length at these time points (Figure 6C). Finally, given that both dendritic and axonal growth rates were linear in males, we compared the growth rates of axons and dendrites across all time points in only the male animals. There was no significant correlation within male animals between axonal and dendritic growth over time (Figure 6D). These results imply that the factors that influence dendritic and axonal growth within the prothoracic ganglion are independent.

## Discussion

This study quantified AN dendritic and N5 axonal growth characteristics in the male and female cricket, *Gryllus bimaculatus*, to better understand general growth patterns of neurons in an adult system after deafferentation. We were surprised to find that the dendritic growth of deafferented AN dendrites was quite different between males and females. Male dendritic growth rates were linear and, on average, surpassed female dendrites in length, while female dendritic growth rates were non-linear, consisting of a burst of growth in the first 3–5 days followed by a plateau. Axonal growth, on the other hand, did not vary by sex. N5 axons did not grow further past the midline in deafferented ganglia as compared to controls but axonal skeletal length did increase linearly after deafferentation. This suggests that axons branched to form more elaborate arbors during the compensatory growth period. We suspect that branching is a major contributor to increasing skeletal length after deafferentation, in both axons and dendrites, but further analysis of branch formation is necessary to confirm this hypothesis (Pfister et al., in preparation). Extraneuropillar sprouting of axons also increased significantly and in a linear fashion in both males and females. These measurements provide a basis of comparison for future studies investigating molecular signals that underlie the growth of AN dendrites and N5 axons after deafferentation.

### Differential effects of unilateral deafferentation on dendrites and axons

It is not entirely surprising that dendrites and axons respond differently to unilateral deafferentation. The AN dendrites are directly deafferented by the loss of their ipsilateral auditory input, whereas the contralateral axons are only indirectly affected by this loss, presumably via the local circuits within the prothoracic ganglion. In addition, axons and dendrites in control animals respond differently to the midline, with dendrites treating it more as a strict border than axons. What is perhaps more interesting is the extent of axonal sprouting observed, given the indirect influence of the deafferentation. Contralateral afferent sprouting does not occur in all deafferented systems. Following unilateral cercal ablation in the cricket and cockroach deafferented giant interneurons in the terminal ganglion and intact contralateral cercal afferents respond by strengthening the weak synapses that already exist between them. Cercal neuron morphology generally remains stable with relatively minor, inconsistent sprouting (Murphey and Levine, 1980; Volman, 1989). In situations where the deafferented interneurons do not have pre-existing contact with contralateral afferents, injury compensation requires more dramatic morphological change. Contralateral sensory sprouting across the midline following auditory deafferentation has been described in several cricket species as well as the locust, *Locusta migratoria* (Pallas and Hoy, 1986; Lakes et al., 1990; Krüger et al., 2011a,b). Limited quantitative measurements of *L. migratoria* contralateral afferent sprouting indicate that the N5 sprouting that we measured in *G. bimaculatus*, although perhaps somewhat more extensive than that seen in the locust, does not appear to be atypical for axonal growth in the insect system (Lakes et al., 1990). After unilateral sensory ablation, input from sensory afferents on the intact side is necessary for dendritic sprouting and compensation in a number of insect systems and is required after deafferentation in the cricket (Murphey and Levine, 1980; Pallas and Hoy, 1986; Volman, 1989; Krüger et al., 2011b). However, it is not clear how deafferented dendrites and contralateral axons are stimulated to grow after deafferentation or whether axons and dendrites signal one another during the growth process. Our measurements indicated that axonal and dendritic sprouting occurred simultaneously but did not provide any hint as to whether axons are attracting dendrites, dendrites are attracting axons or if there is a third party at work.

The N5 growth that we observed crossing the midline is consistent with that previously reported (Schmitz, 1989). The distance that N5 axons extended past the midline and the length of the longest axon in controls did not change after deafferentation. However, N5 skeletal length did increase after deafferentation, indicating that N5 axons must have increased their total length by means of additional axons crossing the midline and/or branching. Although N5 growth has been observed in the adult after larval deafferentation (Horch et al., 2011), N5 extraneuropillar growth has not before been described or quantified after deafferentation in adult crickets. N5 extraneuropillar growth is of particular interest because these axons emerging from the middle region of the claw appear to be in close proximity to AN dendrites, while the axons extending from the anterior-medial portion of the claw across the midline are not in a position to physically interact with sprouting AN dendrites (cf. Figure 1E). Because of the dense and intricate N5 axonal morphology, and because all N5 axons have been backfilled, it is impossible to determine from where exactly the extraneuropillar sprouting originated within N5. However, the compensatory axonal growth extended out, or through, the region of N5 known to contain both low (5 kHz) and ultrasound specific afferents which would appropriately excite AN-1 an AN-2, respectively, (Imaizumi and Pollack, 2005).

The amount of extraneuropillar growth varies extensively among animals (Figure 5B). Given that the formation of synapses is thought to stabilize axon branches (Ruthazer et al., 2006), perhaps stochastic differences in the rate of contact between axons and dendrites, and thus in synapse formation, results in highly variable growth profiles for axons in different animals. Additional auditory interneurons integral to this circuit, such as the Omega neuron, also show compensatory dendritic growth upon unilateral deafferentation (Schildberger et al., 1986). The formation of synapses with post-synaptic partners other than the ANs likely contributes to the overall variation in N5 growth as well, although this has not been studied. Regardless, it is logical to think that the amount of extraneuropillar growth might correlate with the variations in the functional recovery of one-eared animals (Schmitz, 1989), but such a hypothesis has never been tested.

Dendrites in the cricket auditory system, on the other hand, do grow extensively and rapidly across the midline both in males and females. Extensive dendritic growth appears to depend on the availability of contralateral afferents. In the cercal system interneurons already share some contact with contralateral afferents, and thus dendritic morphologic change is generally very subtle after deafferentation, although scattered dendritic sprouting across the midline has been reported for the cricket *Acheta domesticus* (Murphey et al., 1975). In the locust flight system, interneurons share synaptic connections with contralateral afferents while flight motor neurons do not. Following single hindwing tegula ablation, unilaterally deafferented motor neurons will send dendrites across the midline, likely to gain input from contralateral afferents, while neither the contralateral afferents nor interneurons in this case undergo major sprouting (Wolf and Büschges, 1997). After bilateral hindwing ablation in the locust, interneurons have lost both ipsilateral and contralateral input resulting in more extensive interneuron dendritic growth as these neurons seek out forewing afferent input (Büschges et al., 1992). AN interneurons in the cricket do not have contralateral afferent connections prior to deafferentation and thus require extensive growth and rearrangement to receive contralateral input as has been demonstrated in different cricket species (Hoy et al., 1985; Pallas and Hoy, 1986; Brodfuehrer and Hoy, 1988; Schmitz, 1989).

Given that previous studies had noted very few dendrites crossing the midline in controls (Hoy et al., 1985; Schildberger et al., 1986; Brodfuehrer and Hoy, 1988; Schmitz, 1989), we were somewhat surprised to find measurable dendritic midline crossing in control ganglia. However, our approximation of the midline as a single line is probably simplistic. In fact, the glia of the midline do have some width, and it is possible that some of the dendritic growth evident in controls would be reduced if the midline width were better understood. Previous research (Schmitz, 1989) qualitatively described that AN growth in females continuously progressed over time but far more slowly than our measurements indicated. AN-2 was previously reported not to have crossed the midline by 3 days after deafferentation and to have only reached maximum growth past the midline at 27 days after deafferentation. Our observations reported here clearly indicate that AN dendrites not only generally crossed the midline by 3 days, but may even have approached maximum growth by 3–5 days after deafferentation particularly in females (Figure 3). Presumably consistent midline identification, the use of fluorescent tracers and three-dimensional confocal images enhanced the ability to detect small changes in growth.

### Sexually dimorphic dendritic growth in response to injury

We were surprised to find such striking differences in AN dendritic growth rates between male and female adult crickets. If our backfills had included only AN-1, which responds best to the calls of conspecifics, this sexual dimorphism might be expected since males are worse at discriminating between calling songs than females are (Pollack, 1982). However, we believe our images consist predominantly of AN-2 dendrites, which are primarily involved in mediating negative phonotaxis to bat ultrasound calls, with very little contribution of AN-1 dendrites. Assuming that we are looking mainly at AN-2 dendrites, one would not necessarily expect to find varying compensatory growth rates between males and females. AN dendrites in females reached their maximum perpendicular extent, formed their longest dendrite, and completed total arbor growth often by 3 days after deafferentation. Growth then stabilized by 5 days after deafferentation in females. Male AN dendrites, on the other hand, grew more slowly and steadily than female dendrites, but eventually they grew on average twice as long, twice as far beyond the midline, and consisted of twice as much skeletal length as AN-2 female dendritic arbors.

Physiological studies in *Teleogryllus oceanicus* show that functional recovery of ultrasound responses occurs between 4 and 6 days after deafferentation, though tuning curves do not reach control levels even by 28 days after deafferentation (Brodfuehrer and Hoy, 1988). The physiological response strength of AN-2 neurons in females increases over time and in correlation with the increasing number of dendrites crossing the midline (Schmitz, 1989). Physiological and behavioral recovery have not yet been compared between males and females. It is unclear whether females require less AN-2 growth, thus forming more efficient connections with the contralateral N5, than males to regain the same strength in physiological response or if greater total dendritic growth allows males to regain stronger AN-2 physiological responses.

What might cause these sexually dimorphic growth rates in response to injury? Hormones are obvious candidates for creating dimorphic dendritic growth responses. Hormones can influence dendritic and axonal structure both in vertebrates and invertebrates (DeVoogd and Nottebohm, 1981; Cooke and Woolley, 2005; Williams and Truman, 2005). In insects, ecdysteroids, which drive metamorphosis and act as sex hormones, are thought to influence dendritic and axonal growth and branching (Bowen et al., 1984; Truman and Reiss, 1988, 1995; Weeks and Ernst-Utzschneider, 1989; Levine et al., 1991; Prugh et al., 1992; Kraft et al., 1998; Matheson and Levine, 1999; Cayre et al., 2000; Williams and Truman, 2005; Horch et al., 2011). For example, during metamorphosis in *Manduca* and *Drosophila*, certain classes of CNS motor neurons dramatically reorganize their dendritic arborizations in response to shifts in hormones (Duch and Levine, 2000; Consoulas et al., 2002). Dendritic growth during metamorphosis appears to be initiated by low Ca^2+^ levels, which stimulate growth cone formation (Duch and Levine, 2000), and these low Ca^2+^ levels are, at least in part, under ecdysteroid regulation (Grünewald and Levine, 1998).

It is possible that the differences in AN male and female growth in the cricket could be attributed to different hormonal environments. Far greater ecdysone titers have been described in adult *Gryllus firmus* female crickets as compared to males, though the exact levels depend on life history choices (Zera et al., 2007). While ecdysone titers have not been compared directly in adult male and female *G. bimaculatus*, it is reasonable to think that similar differences might exist between males and females in this species (A. Zera personal communication). Furthermore, it is possible that the same hormonal stimulus could influence male and female neuronal morphology in very different ways, as has been demonstrated in mammals (Cambiasso et al., 1995, 2000; Carrer et al., 2005). This indicates that male and female neurons can have inherent differences that may be due, at least in part, to sex chromosome-related gene expression as well as the developmental hormonal environment (Hutchison, 1997; Agate et al., 2003; Dewing et al., 2003). Unlike systems of dramatic sexual dimorphism, we presume that AN-2 reorganization is meant to accomplish the same goal in both males and females: that of regaining the ability to localize bat ultrasound. However, it is not known whether females and males recover AN-2 physiological function at the same time or if the amount of AN-2 growth corresponds to the extent of physiological recovery in either males or females.

AN-2 is tuned to respond to pitches higher than 15 kHz and thus is often thought of as the “bat ultrasound detector.” If indeed the rapid dendritic growth in females demonstrated here is due predominantly to AN-2 dendritic growth, and presuming that this growth corresponds to more rapid functional recovery, why might females need to recover ultrasound detection more rapidly than males? It is possible that females have a higher risk of encountering predatory bats as females spend more time flying at night while searching for males, who are singing in front of their burrows in the ground. Alternatively, it is possible that AN-1 dendrites principally contribute to this sexually dimorphic response. Though both males and females need to respond to conspecific calls, it seems reasonable that females might need to recover this ability more rapidly than males. Though we feel confident that the majority of the dendrites measured in our images are AN-2 dendrites, it would require physiological methods to cleanly document the sexually dimorphic recovery of function in both AN-1 and AN-2 separately.

### Implications for the search for compensatory growth triggers

One of the reasons to carefully quantify dendritic and axonal growth after deafferentation is to try to gather hints about what types of factors might be triggering the unusual compensatory growth in the cricket. Given that N5 axons and AN dendrites grow so differently, it is unlikely that there is a single trigger that induces these changes. In fact, the complete lack of correlation between dendritic and axonal growth strongly implies that separate sets of factors influence axonal and dendritic growth independently. It is likely that a complex milieu of factors is created upon deafferentation, which separately instruct or guide axonal and dendritic growth, and we have some evidence that multiple factors are indeed regulated upon deafferentation (Horch et al., 2009). Furthermore, the results presented here indicate that these factors might interact with hormones or other sex-dependent factors to influence dendritic growth. At a simplistic level, axonal and dendritic growth after deafferentation can be classified into four stages (1) midline crossing; (2) process extension; (3) process elaboration or branching; and (4) synapse formation. These stages are not necessarily discrete nor are they likely to occur in strict chronological order.

Midline regulation is a fundamental component of neuronal organization during development and involves well-conserved molecular mechanisms from worms and flies to mammals. Recent experiments have found that many of the factors that were first described as axon guidance factors also influence the guidance of dendrites (Furrer et al., 2003, 2007; Mauss et al., 2009; Hocking et al., 2010; Teichmann and Shen, 2011). Preliminary evidence indicates that the midline regulator protein, Slit, and the Slit receptor, Robo, do exist in the cricket *G. bimaculatus* (unpublished observations), though no evidence has yet confirmed whether Slit is expressed at the midline. The family of Slit proteins can persist in the adult CNS, as has been demonstrated in rats but the levels and areas of expression during development generally change once the animal reaches adulthood (Marillat et al., 2002). It is possible but not certain that AN dendrites are overcoming a midline barrier as they cross the midline after deafferentation in the adult. The process by which neurons in an adult system are allowed to cross a molecular barrier deserves further study, and understanding this process could contribute to our general understanding of the inhibition of neuronal growth after injury, which is frequently the outcome in most other animal systems.

Though process extension, branching, and subsequent synapse formation are likely influenced by a rich variety of factors, the semaphorin family of neuronal guidance molecules, in theory, could influence all these stages. Semaphorins were first described as chemorepellents for axons (Luo et al., 1993), though they have subsequently been shown to influence the guidance, outgrowth, branching, and targeting of dendrites as well (Polleux et al., 2000; Fenstermaker et al., 2004; Shelly et al., 2011; Sweeney et al., 2011). Semaphorins have also been shown to influence synapse formation during development (Godenschwege et al., 2002; Ding et al., 2011). While the post-developmental roles for semaphorin are less clear, there is good evidence that semaphorin expression levels are regulated after injury in adult mammals (De Winter et al., 2002; Ara et al., 2004; Hashimoto et al., 2004; Kopp et al., 2010). Semaphorin 2a (*Sema 2a*), a secreted semaphorin, has been identified in *G. bimaculatus* (Maynard et al., 2007) and is expressed in the prothoracic ganglion (unpublished observations). Investigation of the differential expression of semaphorins, as well as additional ligands and receptors, will shed light on the mechanisms responsible for this compensatory phenomenon within the adult cricket auditory system.

Research on compensatory neuronal growth in adult crickets is still limited, but the field cricket provides a promising system to better understand fundamental dendritic and axonal growth characteristics that might offer insight into the general mechanisms that trigger neuronal growth in adults. In addition, more strict attention to the possible differences between male and female nervous system function, injury response and recovery at the cellular level, and the interaction of these processes with hormones will likely reveal new insights into how neuronal systems recover from injury.

### Conflict of interest statement

The authors declare that the research was conducted in the absence of any commercial or financial relationships that could be construed as a potential conflict of interest.
